# Analyzing the potential of neuronal pentraxin 2 as a biomarker in neurological disorders: A literature review

**DOI:** 10.3934/Neuroscience.2024031

**Published:** 2024-12-24

**Authors:** Ubaid Ansari, Jimmy Wen, Burhaan Syed, Dawnica Nadora, Romteen Sedighi, Denise Nadora, Vincent Chen, Forshing Lui

**Affiliations:** California Northstate University College of Medicine, USA

**Keywords:** neuronal pentraxin 2, synaptic plasticity, Alzheimer's disease, Parkinson's disease, schizophrenia, neuropathic pain, epilepsy, amyotrophic lateral sclerosis

## Abstract

Neuronal pentraxin 2 (NP2) plays a significant role in synaptic plasticity, neuronal survival, and excitatory synapse regulation. Emerging research suggests that NP2 is implicated in the pathogenesis of various neurological disorders, including neurodegenerative diseases, neuropsychiatric disorders, and neuropathies. This literature review extensively analyzes NP2's role in these conditions, thereby highlighting its contributions to synaptic dysfunction, neuroinflammation, and neurotoxic protein aggregation. In Alzheimer's and Parkinson's diseases, NP2 is linked to amyloid-beta aggregation and dopaminergic neuron degeneration, respectively. Additionally, altered NP2 expression is observed in schizophrenia and bipolar disorder, thus suggesting its involvement in synaptic dysfunction and neurotransmitter imbalance. In neuropathic pain and epilepsy, NP2 modulates the synaptic plasticity and inflammatory responses, with altered levels correlating with disease severity. Furthermore, NP2's involvement in amyotrophic lateral sclerosis (ALS) and frontotemporal dementia (FTD) emphasizes its broad impact on neuronal health. Understanding NP2's multifaceted roles may reveal novel therapeutic targets and improve the clinical outcomes for these neurological disorders. Though the precise role of NP2 remains uncertain, its clinical potential and initial findings justify further investigations into neuronal pentraxins and other related neuroproteins.

## 1. Introduction

Neurological disorders are diseases that affect the brain, spinal cord, and nerves, which make up the central and peripheral nervous systems [1]. These disorders encompass broader categories of human diseases such as neurodegenerative diseases, neuropsychiatric disorders, and neuropathies. Understanding the molecular and cellular mechanisms that underly these diseases is crucial to develop effective therapeutic strategies. Among the various molecular players implicated in neuronal damage, neuronal pentraxin 2 (NP2), a member of the pentraxin family of proteins, has garnered significant attention for its potential role in the pathogenesis of these disorders. NP2 is primarily known for its involvement in synaptic plasticity, neuronal survival, and the regulation of excitatory synapses [2]. It is secreted by neurons and glial cells and can bind to various synaptic proteins, thus facilitating synaptic remodeling and plasticity [2]. Moreover, recent studies have indicated that NP2 may play a critical role in the pathological processes which underlie various neurological human diseases. These findings suggest that NP2 can contribute to the disease pathogenesis through mechanisms such as synaptic dysfunction, neuroinflammation, and the formation of neurotoxic protein aggregates [2]. The scope of NP2's influence spans a wide array of conditions, including neurodegenerative diseases such as Alzheimer's and Parkinson's, neuropsychiatric disorders such as schizophrenia and bipolar disorder, and other neurological conditions such as neuropathic pain, epilepsy, amyotrophic lateral sclerosis (ALS), and frontotemporal dementia (FTD).

NP2 is a secreted protein involved in synaptic plasticity and neuronal survival, with emerging evidence suggesting its significant role in various neurodegenerative and neuropsychiatric disorders. In Alzheimer's disease, NP2 is implicated in amyloid-beta aggregation and synaptic loss, thus contributing to cognitive decline [3]. Similarly, in Parkinson's disease, NP2 has been associated with dopaminergic neuron degeneration and the accumulation of alpha-synuclein aggregates, which are hallmarks of the disease [4]. Beyond neurodegeneration, NP2 is also linked to neuropsychiatric disorders. An altered NP2 expression has been observed in schizophrenia and bipolar disorder, thus indicating a role in synaptic dysfunction and neurotransmitter imbalance [5]. Additionally, NP2 is involved in neuropathic pain by modulating synaptic plasticity and inflammatory responses in the central nervous system [6]. In epilepsy, NP2 expression is altered in response to the seizure activity, thus suggesting its role in the pathophysiology of epilepsy [7]. Furthermore, NP2 has been implicated in ALS and FTD, where it may contribute to motor neuron degeneration and tau pathology, respectively [8]. In these diverse conditions, the multifaceted involvement of NP2 underscores its potential as a therapeutic target to mitigate disease progression and to improve the clinical outcomes.

Despite the growing body of evidence linking NP2 to neurodegenerative disease pathogenesis, the precise mechanisms by which NP2 influences these processes remain incompletely understood. This literature review aims to comprehensively analyze the current research on NP2, thus highlighting its potential contributions to the development and progression of neurodegenerative diseases. By synthesizing findings from various studies, this review seeks to elucidate the multifaceted roles of NP2 in neurodegeneration and explore its potential as a therapeutic target to mitigate disease progression.

## 2. Review

### 2.1. Neuronal pentraxin's role in Synaptic Plasticity and Remodeling

The central nervous system (CNS) excitatory neurons express three main pentraxins: neuronal pentraxin 1 (NP1), NP2, and the neuronal pentraxin receptor (NPR). NP1 and NP2 form oligomers tethered to the plasma membrane by the NPR. The NP complexes bind and cluster α-amino-3-hydroxy-5-methyl-4-isoxazolepropionic acid (AMPA) glutamate receptors and are believed to contribute to several forms of synaptic plasticity and remodeling [9]. The levels of the NP1, NP2, and NPR complex are increased and exocytosed at the excitatory synapses on parvalbumin interneurons (PV-INs) in a diurnal and circadian fashion, thus suggesting a dynamic and behavior-linked process in synaptic refinement [10]. Levels of NP2 have been shown to decrease in response to sleep deprivation and can be monitored by measuring NP2 in the cerebrospinal fluid (CSF) [9]. Additionally, a reduction in NP2 within the CSF can indicate excitatory synapse and inhibitory circuit dysfunction, which is commonly seen in neurodegenerative disorders.

NP2, also known as neuronal activity-regulated pentraxin, is an immediate-early gene that has been linked to synaptic plasticity by aggregating excitatory receptors in the CNS [2]. This effect has been associated specifically with AMPA excitatory synaptogenesis through colocalizing and clustering with GluR1, GluR2, and GluR3 [2]. The NPR is suggested to share a direct expressive pattern with NP2, with NPR knock-out HEK293 cells showing a decrease of NP2 expression by 40% and conversely, a 2-5x increase in NP2 levels with NPR overexpression [11]. Additionally, NPR knock-out cells decreased the levels of NP1 and NP2 at the synapse and has been speculated to stabilize pentraxin on the postsynaptic membrane [11]. NPR, NP1, and NP2 have been found to colocalize to hippocampal neurons and GLuR4 synaptic recruitment. NP2 appears to be particularly important to strengthen the GluR4 excitatory synapse response on PV-INs [9]. However, NP2 levels decrease as the hippocampal neuron-glia co-cultures mature, thus suggesting that NP2 plays a larger role during the early development of the synapse [12],[2]. Interestingly, the greatest aggregation appears to be when NP1 and NP2 are co-expressed [2].

NP2 shares a bidirectional relationship with the brain-derived neurotrophic factor (BDNF), with BDNF inducing NP2 even without neuronal activity. In the synapse, NP2 attaches onto pre- or postsynaptic membranes with the assistance of perineuronal nets (PNNs) via calcium-dependent binding [2]. Cho et al. utilized HEK293 cell cultures and found that the Tumor Necrosis Factor-a Converting Enzyme (TACE) cleaves the N-terminal transmembrane domain of NPR and allows it to cluster with NP2 and AMPA receptors [13]. TACE is upregulated in response to inflammatory conditions and plays an important role in regulating cytokine and growth factor levels during the immune response [14]. Thus, a current proposed mechanism of glutamate excitotoxicity protection is via the removal of AMPA receptors from the synaptic membrane by the clustered NPR, NP2, and AMPA complex.

Moreover, NP2 has been reported to bind to C1q and regulates the classical complement pathway (CCP), specifically in the CNS. NP2 knock-out mice have shown increased CCP levels, thus resulting in the removal of excitatory synapses in the CNS. Conversely, overexpression of NP2 reduced the neuronal damage in P301S mice. C1q, CCP, and other complement components are up-regulated in many neurodegenerative disorders and are correlated with synapse loss and neuronal damage [9]. Thus, this supports the idea that NP2 is highly involved in the regulation of synapse formation, removal, and maintenance.

The exact role and mechanism of action of NP2 are not fully characterized, though they have been associated with essential synaptic plasticity and synaptogenesis processes via AMPA trafficking and mGLuR1/5-dependent long term depression (LTD) [2]. Additionally, the majority of NP2 research has been conducted in neurons, and other downstream effects on other CNS cells may be occurring as well [15].

### 2.2. Neurodegenerative Diseases (Alzheimer's, Parkinson's)

Alzheimer disease is characterized by neurofibrillary tangles and amyloid-beta peptide accumulation. These aggregates initially occur in areas involved in memory, such as the entorhinal cortex and hippocampus, and then spread to other areas of the cerebral cortex, such as the medial temporal lobe. Changes in behavior, learning, reasoning, and language can occur over time [16],[17]. When triggered by oxidative stress, neuroinflammation is also another mechanism proposed for the pathogenesis of the disease. The inflammatory cascade triggered by damage due to the accumulation of peptides, such as amyloid-beta plaques, can cause degeneration in neurons and synaptic strength, thus leading to a cognitive decline [18]. Synaptic damage and loss have been marked in post-mortem brain tissue in people diagnosed with this disease, and further studies have even shown that specific synaptic changes other than loss, such as fewer or modified mitochondria, can occur in the disease progression [19]–[21]. These processes relate back to the role of the neuronal pentraxin protein family, specifically their role in the synapse functionality, neuroprotection, and inflammatory responses.

The neuronal pentraxin family of proteins hold an important functionality in the progression of Alzheimer's disease. As mentioned before, specific hallmarks of the disease, such as neuroinflammation and synaptic damage, are influenced by the role of neuronal pentraxin proteins [22]. Specifically, NP2 displays synaptic plasticity abilities that are important in the pathogenesis of the disease. Although the exact role of NP2 in the setting of neuroplasticity has not been elucidated, the proposed mechanisms suggest that NP2 influences the aggregation of AMPA receptors at the pre- and postsynaptic membranes, thereby regulating the formation and stability of synapses [23]. NP2's role in neuroinflammation, especially its function of clearing extracellular pathogens, toxins, and synaptic debris, can further link the protein and Alzheimer's pathology [24]. Due to its potential relationship with the disease, it is unsurprising that NP2 in the CSF is currently being evaluated as a potential prognostic biomarker for Alzheimer's disease. Reduced levels of NP2 in the cerebrospinal fluid of patients with the disease were associated with cortical atrophy, as well as changes in already established biomarkers, such as the levels of amyloid-beta and neurofilament light chain peptides [25],[26]. This suggests the importance of NP2 in the progression and detection of Alzheimer's, and further studies would be instrumental in finding its exact role for detection and therapeutic purposes.

Another common neurodegenerative disease with a potential relation to NP2 is Parkinson's disease. This disease is characterized by the damage and destruction of dopaminergic neurons in the substantia nigra pars compacta, neuroinflammation, and aggregates of misfolded alpha-synuclein proteins [27],[28]. These aggregates appear as intra-cytoplasmic Lewy bodies and neurites, which are a hallmark of the disease. Neuroinflammation along with the aggregates can cause neuronal damage and are responsible for the symptoms seen in this disease, specifically bradykinesia, rigidity, resting tremors, and sleep disturbances [29]. Additionally, understanding the role of the neuronal pentraxin family of proteins in this disease can lead us to a better understanding of their function in a healthy brain.

Although NP2's role in Parkinson's is less defined than in Alzheimer's, many of the same proposed mechanisms can be applied in this disease process as well. Recent studies have shown the effect of synaptic dysfunction and the effect of neurotransmitter release in the setting of Parkinson's. It can be assumed that the neuronal pentraxin family could have a role in this pathogenesis due to their role in synaptic upkeep and regulation. Interestingly, novel studies have shown that the NP2 gene is the most highly upregulated gene in the substantia nigra of patients with Parkinson's [4],[30]. Additionally, NP2 accumulation was found in Lewy body and neurites, along with associated finger-like projections or bridges between the Lewy bodies. This could perhaps implicate NP2 in the growth of Lewy bodies, and thus the progression of Parkinson's disease. Despite the down regulation of alpha-synuclein gene expression, the upregulation of NP2 can also point towards its connection to Parkinson's disease [4]. Orexin producing neuron destruction, which is also implicated in Parkinson's, displays a relatively high expression of NP2 and leads to sleep disturbances, which are common in Parkinson's patients [31]. Finally, with dopaminergic neurons being more susceptible to excitotoxicity due to the AMPA receptor activation, and thus leading to cell death, NP2's role in the destruction of these neurons is further implicated in the disease progression [4],[32]. Further studies have to be performed in order to ascertain the exact causality and relationship of NP2 with Alzheimer's, Parkinson's, and other neurodegenerative diseases. Understanding this relationship can potentially lead to new therapeutic targets and a greater understanding of these diseases, as well as the role of the neuronal pentraxin family in the body.

### 2.3. Neuropsychiatric Disorders (Schizophrenia, Bipolar Disorder)

Schizophrenia is a heterogenous neuropsychiatric disorder that often presents in early adulthood and is marked by sensory hallucinations, delusions, social withdrawal, decreased volition, and an impaired cognitive function [33]. Current understandings of the illness have yet to sufficiently explain its etiology, but favored views suggest a combination of polygenic factors and environmental epigenetic influences [33]. The dysregulation of dopaminergic pathways is believed to be a major precipitant in the psychotic manifestations of schizophrenia, though other mechanisms such as a glutamatergic impairment and a diminished activity of N-Methyl-D-Aspartate (NMDA) receptors have also been suggested to explain the constellation of symptoms associated with the disease [34],[35]. Abnormal synaptic activity, synapse loss, and neuroinflammation have all been found to be important features in the pathophysiology of schizophrenia [36]–[38]. Similarly, in bipolar disorder, there is evidence that glutamate-mediated synaptic plasticity through AMPA receptors and the altered expression of synapse-related genes may be relevant pathomechanisms [39],[40]. Bipolar disorder is a chronic psychiatric disorder classically characterized by the dysregulation of mood with fluctuating states of mania, hypomania, and depression [41]. As such, schizophrenia and bipolar disorder may each represent potential targets of analysis through biochemical processes that involve NP2.

A growing body of research has found evidence of altered NP2 expression in association with schizophrenia. As previously discussed, NP2 plays a role in modulating the complement activation within the CNS by preventing neuroinflammation and microglia-mediated synaptic loss, which is a pathway that has also been implicated in schizophrenia-associated genes [42],[43]. Additionally, the neuropsychiatric impact of NP2 loss has been evaluated using mouse models, with *Np2* double-knockout mice demonstrating stronger secondary fear responses, an increased social isolation, and a greater propensity to acquire schizophrenia-like behavioral domains after stress induction compared to wild-type mice [10]. One meta-analysis of microarrays compared samples from living, human-induced pluripotent stem cells (hiPSCs), and post-mortem brain tissue from individuals with schizophrenia against healthy controls, as a means of mimicking neural conditions in early diagnosis (hiPSCs) and chronic disease (post-mortem) in schizophrenia [44]. This investigation revealed a significant downregulation of *Np2* in both the post-mortem brain tissue and the hiPSCs derived from living individuals with schizophrenia, thus suggesting that the NP2 reduction plays a role in both early and late-stage disease [44]. This alteration in the brain NP2 was not found in a similar microarray analysis of the peripheral tissues in individuals with schizophrenia [45].

An analysis of two independent cohorts of individuals with new-onset schizophrenia against healthy, age-matched controls revealed significant decreases in the NP2 levels in the CSF of the schizophrenia groups when compared with the control groups [10]. Furthermore, while NP2 levels within the schizophrenia cohorts could not be associated with the severity of positive/negative symptoms, the lower levels were correlated with a decrease in the ideational fluency [10]. A more recent study conducted at Xinxiang University in 2024 found that plasma NP2 levels were significantly decreased in individuals with schizophrenia when matched against healthy controls; however, this difference was notably no longer existent during an 8-week follow-up, during which the schizophrenia group underwent an antipsychotic pharmacotherapy [5]. This could suggest that one mechanism through which antipsychotics treat schizophrenia is via a suppression of neuroinflammation, and thus NP2 [5]. In contrast, researchers who studied schizophrenia patients during acute psychotic episodes have observed increased plasma NP2 levels compared to those of healthy individuals, with increased levels actually being associated with lower scores on the Positive and Negative Syndrome Scale (PANSS), thus indicating a greater disease burden [46].

Alterations in NP2 expression in schizophrenia have also been localized to the cortical brain regions involved in visuospatial working memory (vsWM), which is often diminished in individuals with the disease [47]. Relative to healthy controls, post-mortem NP2 mRNA levels in the schizophrenia group showed reductions throughout all four cortical regions of the vsWM network, thus suggesting the impaired activity of pyramidal neurons and the alterations in parvalbumin (PV) and somatostatin (SST) inhibitory neurons observed in schizophrenia [47]. Additionally, increased plasma NP2 levels have been significantly correlated with increased Category Fluency Test scores (a measure of cognitive ability) and decreased white matter volumes in the superior temporal gyrus of individuals with schizophrenia, which may elucidate the involvement of NP2 in the progression of the disease [5].

Although there is evidence that NP2 may be a protein of significance in bipolar disease, the investigation of its role in the pathophysiology of the illness has been less conclusive. In one case-control study, there was no significant difference in the NP2 levels in cerebrospinal fluid between bipolar and healthy control individuals, nor when comparing levels during a dysthymic episode to baseline in an individual with bipolar disease [48]. However, while not significant, the difference in levels was still discernible from the time of the acute mood episode to a one-year follow-up, which is similar to the trend observed with NP2 in individuals with Alzheimer's disease [48]. Moreover, other research that involved a postmortem synaptic analysis of proteins in the dorsolateral prefrontal cortex in individuals with schizophrenia and bipolar disease found a marked reduction in the NP2 levels when compared with matched controls [49]. Notably, NP2 was shown to be among the most heavily reduced synaptic peptides in individuals with schizophrenia, and bipolar disease to a lesser extent [49]. In addition, bipolar disease has previously been linked to a similar region of the genome as the locus of the *Np2* gene on chromosome 7q21.3-22.1, though the association still remains relatively unclear [50]. Of course, while the aberrant NP2 activity and expression may be potent avenues through which to analyze schizophrenia and bipolar disorder, more research must ultimately be performed to determine both its clinical usage as a biomarker and its role in the pathophysiology of these diseases.

### 2.4. Neuropathic Pain

Neuropathic pain has been associated with imbalances between excitatory and inhibitory somatosensory signaling, changes in ion channels, and variations in the modulation of pain signals within the CNS [51]. Additionally, it has been associated with an impaired quality of life and is often poorly managed, thus resulting in an increased use of drug prescriptions as well as visits to healthcare providers. Around 7–8% of adults have pain with neuropathic characteristics, which includes allodynia (pain that occurs in response to stimuli that are typically not painful), hyperalgesia (increased response and sensitivity to feeling pain), and paresthesia (a burning or prickling sensation that is usually felt in the hands, arms, legs, or feet, but can also occur in other parts of the body) [52]. In addition, the International Association for the Study of Pain (IASP) has now defined neuropathic pain as “pain caused by a lesion or disease of the somatosensory nervous system” [53]. Recently, in a sciatic nerve transection (SNT) model, NP2 knock-out (KO) mice exhibited an exaggerated microglia/macrophage response compared to wild-type mice. These KO mice displayed cognitive inflexibility and an addictive behavior, which are linked to abnormal reactivity to novel stimuli, thus suggesting that NP2 is associated with cognitive impairment and may be a potential biomarker for the development of neuropathic pain [54].

In a 2021 study using a mouse model, Wang et al. investigated the effects of neuropathic pain on cognitive function, thereby focusing on the down-regulation of neurotrophic factors such as NP2 in the cortex and hippocampus [6]. NP2 plays a critical role in synaptic plasticity and cognitive function, thus serving as a synaptic organizer and stabilizer of AMPA receptors, which are essential for excitatory synapse function. The study found that the expression of NP2 in the cortex and hippocampus of the spared nerve injury cognitive impairment group was significantly decreased compared to the sham group. Furthermore, behavioral experiments with NP2 KO mice demonstrated that the deletion of NP2 could lead to increased cognitive dysfunction and anxiety-like behaviors. The study highlights that chronic neuropathic pain results in a significant down-regulation of NP2. Consequently, reduced NP2 levels impair the synaptic plasticity, thus leading to the cognitive deficits observed in neuropathic pain conditions [6].

The study concluded that NP2 may possibly be used as an indicator of neuropathic pain-induced cognitive dysfunction and may play a role in the inflammatory response regulation, thus contributing to the cognitive impairment induced by chronic pain. NP2's broad expression and its involvement in synaptic plasticity and neuroprotection highlight its potential as a therapeutic target for cognitive impairments associated with neuropathic pain and other neurological disorders [2].

### 2.5. Epilepsy

Epilepsy is a neurological disorder characterized by recurrent, unprovoked seizures [55]. These seizures result from an abnormal electrical activity in the brain, which can cause various symptoms depending on the part of the brain that is affected. Epilepsy can affect people of all ages and can vary in severity from mild to severe [55]. Cognitive impairment occurs more often among patients with epilepsy. The risk factors include the age of onset, the seizure type, frequency, and the duration of the seizures [56]. Unfortunately, there is no diagnostic biomarker for epileptic patients with a cognitive impairment. NP2, which is important for neurotransmission and synaptic plasticity, was studied for its potential as a biomarker [7]. The study was conducted at the First Affiliated Hospital of Fujian Medical University between January 2020 and December 2021. The study involved 74 epilepsy patients with normal cognitive function, 37 with cognitive dysfunction, and 30 healthy controls [7]. Cognitive function was assessed using the mini-mental state examination (MMSE), and the serum NP2 levels and electroencephalogram (EEG) signals were analyzed. The results showed lower serum NP2 levels in patients with cognitive dysfunction, who also had the lowest MMSE scores [7]. In these patients, the NP2 levels were positively correlated with the MMSE scores and were negatively correlated with the epilepsy duration and the EEG slow wave/fast wave frequency ratio. Thus, serum NP2 is a potential biomarker to diagnose cognitive impairment in epilepsy patients [7].

NP2 is also upregulated in epileptic mice. Xing et al. conducted in vitro experiments with Mg2+ free medium in which they used a mouse model of epilepsy induced by kainic acid [57]. These researchers found that a NP2 knockdown reduced the seizure frequency and duration, decreased the EEG amplitude, and improved the learning and memory abilities. Silencing NP2 mitigated epilepsy-induced brain damage and reduced the neuron apoptosis, as shown by the decreased expression of pro-apoptotic markers and the increased anti-apoptotic Bcl-2 expression. Additionally, NP2 knockdown inhibited GluA1 phosphorylation, reduced GluA1 membrane expression, and reversed the decline in PSD95 expression seen in epilepsy [57]. These findings suggest that NP2 is a promising target for epilepsy treatments, as its silencing alleviates brain damage and enhances cognitive function.

Perinatal hypoxia-ischemia (HI) is one of the leading causes of cerebral palsy and it often results in epilepsy [58]. HI triggers excitotoxicity, in which the glutamate receptors in the brain are overstimulated, thus leading to cell death in neurons and glial cells. The immature brain's unique vulnerability to HI influences the pattern of damage and results in neurological disorders such as cerebral palsy and epilepsy. An HI-induced brain injury progresses over days to weeks, thus suggesting a "therapeutic window" for intervention. Apoptotic pathways play a significant role in the HI pathophysiology, and new protective strategies include neuronal growth factors and the inhibition of apoptotic pathways. Additionally, recent findings draw attention to another member of the pentraxin family of proteins called NP1, which is a protein induced by HI. Interestingly, it can be neuroprotective when silenced, thus suggesting that it could be a potential target to prevent an HI injury. NP1's interaction with glutamate receptors implicates it in the excitotoxic cascade, and targeting these delayed events could provide therapeutic opportunities to mitigate long-term neurological disabilities [58].

### 2.6. Amyotrophic Lateral Sclerosis and Frontotemporal Dementia

Amyotrophic lateral sclerosis (ALS) is a progressive neurological disease that leads to the deterioration of the upper and lower motor neurons in the brain and spinal cord [59]. It is characterized by asymmetrical, bilateral limb weakness, stiffness and ultimately paralysis [60]. In contrast, frontotemporal dementia (FTD) is a heterogenous group of neurodegenerative conditions that cause the gradual loss of nerve cells in the frontal and temporal lobes of the brain [61]. This produces language and motor skills deficits and impairments, as well as promotes changes in behavior. However, the lack of comorbidities and diagnostic tools makes early detection especially difficult, thus exacerbating management for an already challenging group of diseases [25]. Additionally, although ALS and FTD are two distinct diseases, they share pathological features, including synaptic biomarkers such as transactive response DNA-binding protein of 43 kDA (TDP-43) [62]. Current studies have shown that NP2, which is a protein involved in excitatory synapse formation, may also play a role in the pathophysiology in the inherited forms of ALS and FTD.

NP2 has shown a great clinical potential as a biomarker of FTD [32]. NP2 complexes with the NPRs at excitatory synapses of pyramidal neurons, thus playing a role in synaptic homeostatic plasticity. Decreased levels of NP2 contribute to synaptic dysfunction, which has emerged as an early pathological event in FTD. Thus, fluid biomarkers of synaptic degeneration might help with the early detection and consistent management of the disease. One study that focused on genetic FTD found that the cerebrospinal fluid NP2 concentration was lower in the symptomatic mutation carriers than in the pre-symptomatic carriers and non-carriers [32]. Additionally, the NP2 concentration corresponded with the disease severity and gray matter volume. Moreover, it predicted declines in verbal fluency and the Clinical Dementia Rating.

In hereditary FTD with mutations in granulin (GRN) and the chromosome 9 open reading frame 72 (c9orf72), which play key roles in neuronal survival and RNA production and transport, symptomatic mutation carriers were observed to have significantly lower levels of NP2 compared to presymptomatic carriers and non-carriers [32]. A study by van der Ende et al. conducted across 16 centers in Europe and Canada included subjects with FTD who had mutations in the GRN, C9orf72, or microtubule-associated protein tau (MAPT) genes. The study included 54 symptomatic mutation carriers, 106 presymptomatic mutation carriers, and 70 non-carriers. The NP2 levels were measured using an enzyme-linked immunosorbent assay (ELISA) and the results showed that when the different mutations groups were analyzed, the GRN and C9orf72 mutation carriers had significantly lower levels of NP2 in symptomatic individuals than the pre-symptomatic and non-carrier counterparts. Additionally, the individuals with symptomatic MAPT mutations had significantly lower levels of NP2 compared to the non-carriers, but not compared to the presymptomatic carriers [32]. Thus, the NP2 levels in the CSF may be a potential biomarker to diagnose and monitor neural circuit dysfunction in genetic FTD.

In the context of ALS, the NP2 levels appear to vary depending on the specific models and stages of the disease. It is well known that the dysregulation of TDP-43 in ALS plays a role in the pathogenesis of the disease and can be seen in more than 90% of ALS patients [63]. However, dysfunction of TDP-43 can also affect the expression of various target genes, including NP2. A study by Hruska-Plochan showed that the levels of NP2 were controlled by TDP-43 binding to its 3′ untranslated region [8]. Additionally, the study showed neurotoxic effects when NP2 was expressed in high levels by the mature neurons. However, when the NP2 levels were lowered, neurodegeneration caused by TDP-43 could be partially rescued [8]. This suggests that NP2 may play a role in mediating TDP-43-induced neurotoxicity. With NP2's involvement in neurodegenerative diseases and its potential role as a biomarker and therapeutic target, further research is needed to fully understand the mechanisms by which NP2 interacts with TDP-43 and contributes to ALS pathology.

## 3. Conclusion

The pentraxin family has gained an increased interest in its potential to be biomarkers for neurological diseases, and understanding their mechanism of action will open up new avenues for therapeutic research. Specifically, NP2 has been linked to several essential processes involved in synaptic plasticity and synaptogenesis. NP2's involvement in clustering AMPA receptors at synapses calls attention to its essential function in synaptic organization and stabilization, thereby directly influencing excitatory neurotransmission and cognitive processes. The dynamic regulation of NP2 in response to circadian rhythms and behavioral states further highlights its significance in maintaining synaptic integrity. These processes and the concentration of NP2 have been linked to the pathogenesis and progression of several neurological disorders included in this review. Additionally, this review synthesized the current literature of NP2's role in the CNS, neurodegeneration, and role as a biomarker and therapeutic target. For neurodegenerative conditions, such as Parkinson's disease, Alzheimer's disease, ALS, and FTD, NP2's involvement in synaptic homeostasis and degeneration positions it as a valuable biomarker for early diagnosis and disease monitoring. Additionally, neuropsychiatric disorders, including schizophrenia and bipolar disorder, demonstrate altered NP2 expressions which correlated with behavioral and cognitive deficits. However, its role in the CNS has not been fully defined and remains an area of research to be further explored in the literature. By understanding NP2's role in synaptic organization and neuroprotection, new therapeutic strategies can be developed to mitigate cognitive impairment in neuropathic pain, epilepsy, and related conditions. As the grasp on the underlying mechanisms of different neurological disorders evolves, research will hopefully uncover new diagnostic and therapeutic methodologies for these challenging diseases. Although the exact role that NP2 plays is still unclear, its clinical potential and preliminary data warrants further research into neuronal pentraxins and other neuroproteins.
